# Application of Edge Computing in Structural Health Monitoring of Simply Supported PCI Girder Bridges

**DOI:** 10.3390/s22228711

**Published:** 2022-11-11

**Authors:** Yi-Ching Lin, Chin-Yu Hsiao, Jian-Hua Tong, Chih-Pin Liao, Shin-Tai Song, Hsin-Chu Tsai, Jui-Lin Wang

**Affiliations:** 1 Department of Civil Engineering, National Chung Hsing University, 145 Xingda Rd., South Dist., Taichung 402, Taiwan; 2 Department of Computer Science and Information Engineering, Hungkuang University, No. 1018, Sec. 6, Taiwan Boulevard, Shalu District, Taichung 433, Taiwan; 3 The Second Maintenance Office, Directorate General of Highways, No. 127, Daquan St., West Dist., Taichung 403, Taiwan; 4 China Engineering Consultants, Inc., Floor 28, No. 185, Section 2, Sinhai Road Da-an Dist., Taipei City 106, Taiwan

**Keywords:** dynamic strains, structural health monitoring, prestressed concrete I girders, flexural rigidity, edge computing

## Abstract

This study proposes an innovative method for structural health monitoring of simply supported PCI girder bridges based on dynamic strain and edge computing. Field static and dynamic load tests were conducted on a bridge consisting of a span with newly replaced PCI girders and numerous spans with old PCI girders. Both the static and dynamic test results showed that the flexural rigidity of the old PCI girders decreased significantly due to deterioration. To improve the efficiency of on-site monitoring data transmission and data analysis, this study developed a smart dynamic strain gauge node with the function of edge computing. Continuous data with a sampling frequency of 100 Hz were computed at the sensor node. Among the computed results, only the maximum dynamic strain data caused by the passage of the heaviest vehicle within 1 min were transmitted. The on-site monitoring results indicated that under routine traffic conditions, the dynamic strain response of the new PCI girder was smaller than that of the deteriorated PCI girder. When the monitored dynamic strain response has a tendency to magnify, attention should be paid to the potential prestress loss or other deterioration behaviors of the bridge.

## 1. Introduction

The development of structural health monitoring (SHM) technology for bridges is a major goal in civil engineering. The SHM of a bridge involves using different types of sensors, such as accelerometers, inclinometers, displacement sensors, strain gauges, load cells, and thermometers, for monitoring the response of the bridge to external stimuli [1,2]. Analysis of the structural system characteristics of the measured structural response enables the real-time detection of structural damage or deterioration for ensuring the structural safety of a bridge. SHM systems generally comprise sensor systems, data acquisition systems, monitoring centers, and signal analysis and diagnosis technology [3]. Compared with the traditional method of wired sensors, wireless sensors are very suitable for structural health monitoring of bridges because of their convenient installation, low maintenance costs and flexible deployment. Therefore, the wireless sensor network (WSN) has become the main development technology for bridge SHM [4,5,6,7,8,9,10,11].

According to the sampling frequency used, SHM can be categorized as static monitoring and dynamic monitoring. In general, in static monitoring, the sampling frequency is relatively low and data acquisition is performed at a specific time interval, such as every 1 s, 1 min, 10 min, or 1 h. The sampling frequency in dynamic monitoring is considerably higher than that in static monitoring. For bridge structures with natural frequencies of vibration between 0.2 and 20 Hz, a sampling frequency of 100 Hz (i.e., 100 data entries acquired per second) is sufficient.

SHM technologies for bridges have been developed for more than 30 years. Unfortunately, the influences of environmental temperature changes on the physical behavior of bridges make it difficult to use common indicators such as bridge vibration frequency, static displacement, and static strain for examining the structural health of bridges [12,13,14]. The structural health of bridges can only be examined through qualitative trend analysis by using various statistical analysis methods [15,16,17]. Suitable “quantitative” indicators have not been identified for real-time bridge safety management [18].

The structural strain of bridges is sensitive to external forces; thus, strain monitoring may be a suitable approach for the SHM of bridges. However, the traditional static strain monitoring method is severely affected by environmental temperature changes. In general, a strain gauge measures the apparent strain, which includes the strain caused by temperature changes and the mechanical strain. Theoretically, the mechanical strain required for structural analysis can be obtained if the temperature-induced strain and apparent strain are known. However, because current commonly used strain gauges, such as resistance strain gauges, vibrating-wire strain gauges, and optical fiber strain gauges are affected by long-time temperature changes, the measured strain exhibits a drift phenomenon, which makes signal analysis extremely difficult [19,20,21,22,23,24]; sometimes, effectively obtaining a reasonable mechanical strain is extremely difficult. Thermal drift results from the characteristics of the hysteresis loop formed by the nonlinear relationship between temperature and strain changes when an external force is absent [22,23,24]. Therefore, even when the temperature change is restored to the original value, the measured strain cannot be restored to the strain’s original value. Recurrent temperature changes of extended duration result in the phenomenon of accumulated deviation in the measured strain (i.e., the drift), which increases the difficulty of mechanical strain analysis.

In recent years, some studies have used dynamic strain devices to measure the strain responses caused by vehicles passing through bridges [25,26,27]. Since the duration of the vehicle-induced strain is very short, the temperature influence can be ignored, and the mechanical stress caused by the vehicle can be reasonably analyzed. More references in this field can be found in recent review papers [28,29].

This study performed field static and dynamic load tests on a bridge consisting of a span with newly replaced PCI girders and numerous spans with old PCI girders to demonstrate the vehicle-induced strains can be used to evaluate the deterioration of the old PCI girders. Subsequently, the dynamic strain gauges were deployed to monitor the relative strain change caused by the dynamic load of the vehicles. In general, a vehicle requires only a few seconds to pass through a prestressed concrete bridge. The temperature can be considered to be constant during this time. Therefore, the relative strain change caused by the passage of a vehicle is only affected by the weight of the vehicle and the flexural rigidity of the PCI girder. Thus, the dynamic strain examined in this study is unaffected by temperature. Since the flexural rigidity of a PCI girder decreases due to deterioration including concrete cracking, prestress loss, etc., the relative strain change exerted by a vehicle passing over the PCI girder can serve as an indicator of the deterioration of the PCI girder.

Dynamic strain measurement requires the use of high-cost data acquisition equipment, and a considerable amount of data must be recorded and transmitted for dynamic strain monitoring. Therefore, on-site dynamic strain monitoring systems have a high workload, making them power-intensive and prone to system instability. Consequently, the on-site application of these systems is difficult. To overcome the problem caused by the need to transmit considerable data generated through dynamic measurement, this study adopted the edge computing technology, which is used in Internet of Things (IoT) frameworks. In edge computing, the data computation and analysis processes for an application are migrated from the central node in the network (cloud server) to edge nodes (the sensor nodes); thus, the sensor nodes only have to transmit key computed data. The main strength of edge computing is that it reduces the load on the network and servers so that each sensor can be directly equipped with a narrowband IoT (NB-IoT) wireless communication module which is a Low Power Wide Area Network (LPWAN) radio technology. This data monitoring process is highly effective and independent and does not require an accompanying host or gateway on site. The combination of the dynamic strain measurement and edge computing can form a simple, straightforward, and effective method for evaluating the degradation of simply supported PCI girders.

The important findings of this paper are as follows:

- The flexural rigidity of the old PCI girders decreased significantly due to deterioration.
- The vehicle-induced strains of old and deteriorated PCI girders were noticeably larger than those of the newly replaced PCI girders.
- This study developed a smart dynamic strain gauge node with edge computing functions to improve the monitoring system efficiency.
- The vehicle-induced strain responses were stable under routine traffic conditions. When the monitored dynamic strain response tends to amplify, attention should be paid to the potential prestress loss or other deterioration behaviors of the bridge.

This paper first presents the results of static and dynamic tests performed on a PCI girder bridge. Secondly, the development of a smart dynamic strain gauge node with edge computing functions is introduced, followed by the field application of the smart dynamic strain gauge node. Finally, based on the results obtained in this study, discussions and conclusions are given.

## 2. Static and Dynamic Tests of a PCI-Girder Bridge

### 2.1. Description of the Bridge

**Figure 1:**
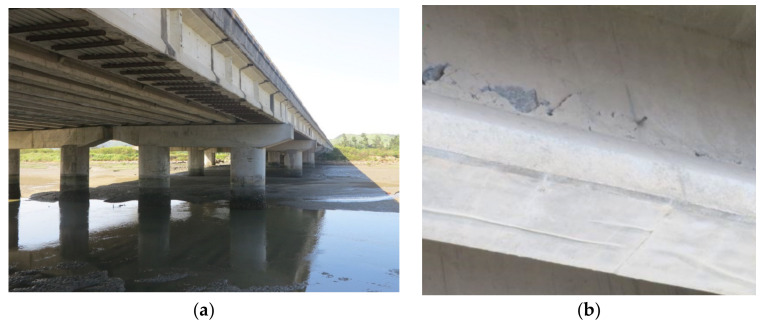
Photograph of the investigated bridge: (**a**) side view; (**b**) cracks along the tendon duct.

The field static and dynamic tests were carried out on a bridge located near the west coast of Taiwan, which was completed in October 1995. Figure 1a shows the photograph of the bridge. Except for the two spans at the both ends of the bridge, which consisted of a 20 m long prestressed concrete I-girder (PCI girder), the remaining eight spans of the bridge consisted of a 40 m long simply supported PCI girder. Because obvious cracks were present along the prestress tendon duct of the seventh northward span of the bridge as shown in Figure 1b, the PCI girder of this span was replaced in October 2018. The cross-sectional dimensions of the new PCI girder were similar to those of the old ones. Thus, the PCI girder of the seventh span could serve as the control group in the vehicle loading test for comparing the differences in the obtained signals between the new PCI girder and the other old and deteriorated PCI girders.

### 2.2. Static Test

**Figure 2:**
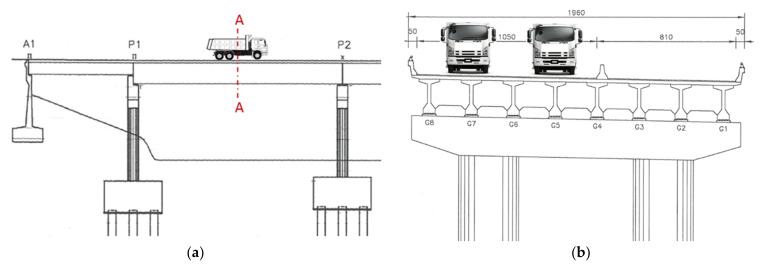
Schematic of the static test: (**a**) elevated view; (**b**) section A-A view.

**Figure 3:**
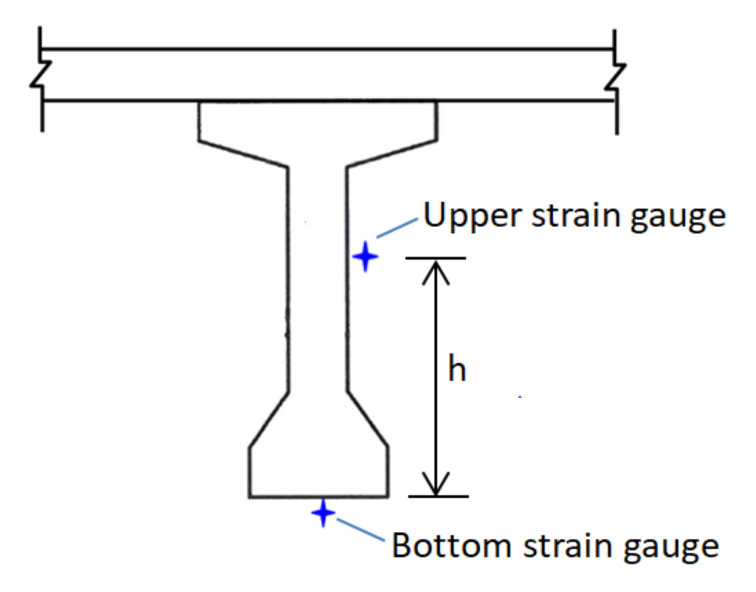
Schematic of the deployment of two strain gauges for the static test.

In the static loading test, two 24-ton vehicles were placed in parallel on the bridge deck at the mid-span of the simply supported PCI girders, as shown in Figure 2. Two strain gauges were installed on the cross section of the midpoint of the G5 girder under the lane where the vehicle passed, as depicted in Figure 3, where the distance between the upper and bottom strain gauges was denoted as h. The sampling time interval of the strain gauge was 5 s. The strain gauge recorded the strain response caused by placing two fixed-weight vehicles over the span.

**Figure 4:**
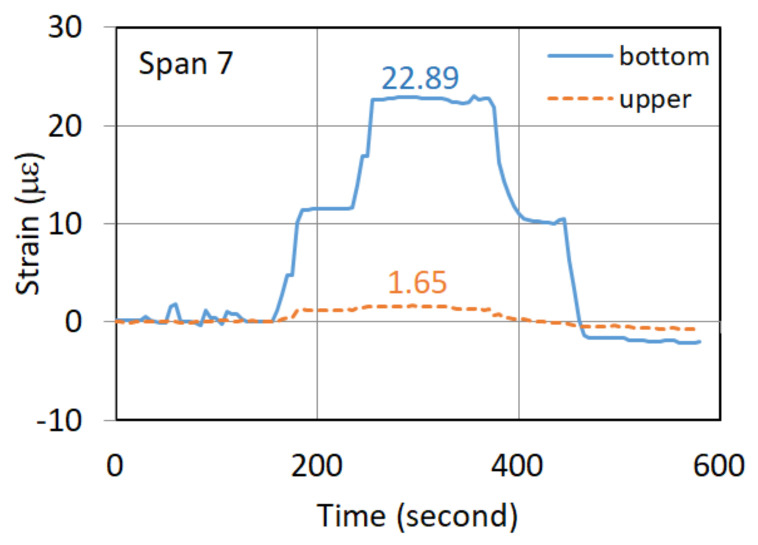
Strain response of the seventh northward span under the static test.

**Figure 5:**
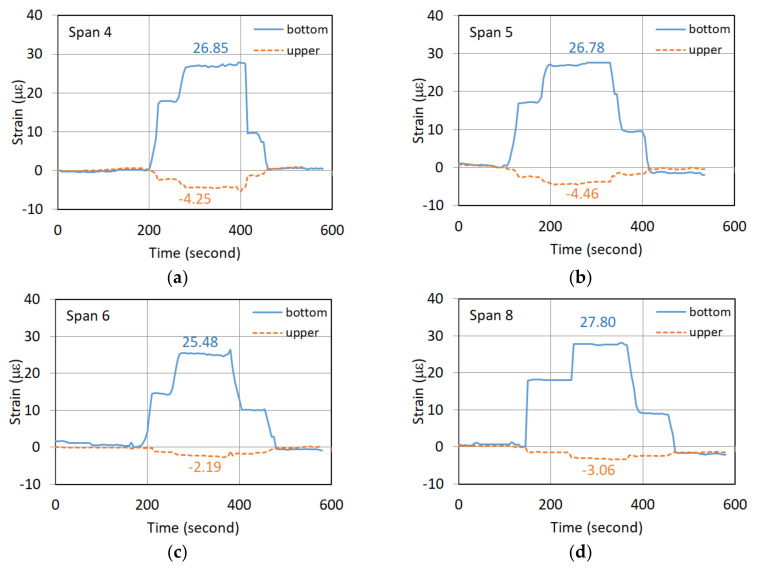
Strain responses of northward spans during the static test: (**a**) Span 4; (**b**) Span 5; (**c**) Span 6; (**d**) Span 8.

The static vehicle test was performed by slowly moving the first vehicle to a designated location at the midpoint of the span, and then moving the second vehicle. These two vehicles were placed in the specific locations and lasted for at least 100 s. Figure 4 shows the recorded strain response of the G5 girder of the seventh northward span in a static vehicle test. The strain responses were identified to be 22.89 and 1.65 µε (microstrain) for the bottom and upper strain gauges, respectively, when both the two vehicles were placed in the specific locations on the span. Figure 5a–d depict the strain responses of the static vehicle test for the fourth, fifth, sixth, and eighth spans in the northward direction, respectively.

Knowing the distance (*h*) between the bottom and upper strain gauges, the measured strains can be used to determine the curvature (κ) at the cross-section where the strain gauges are installed as follows:

(1) $$\kappa =\frac{\left(\varepsilon_{b}-\varepsilon_{u}\right)}{h}$$

The curvature (κ) can also be related to the moment (M) acting on the cross-section and the flexural rigidity (*EI*) as follows:

(2) $$\kappa =\frac{M}{EI}=\frac{\left(\varepsilon_{b}-\varepsilon_{u}\right)}{h}$$

Since the vehicle load applied to each span was the same in the static test, the moment (M) acting on the investigated cross-section of the simply supported PCI girder can be regarded as a constant. Thus, *EI/M* represents the relative magnitude of *EI* for each span, which can be calculated using Equation (3).

(3) $$\frac{EI}{M}=\frac{h}{\left(\varepsilon_{b}-\varepsilon_{u}\right)}$$

Table 1 summarizes the static test results. In Table 1, the second and third columns give the strain responses of the bottom and upper strain gauges, respectively, obtained from the static vehicle test. With the known distance (h) as listed in the fourth column of the table, the calculated EI/M for each span was given in the fifth column. As shown in Table 1, the magnitude of EI/M of the newly replaced girder of the seventh span was higher than those of the fourth, fifth, sixth, and eight spans. As listed in the last column of Table 1, the EI/M ratios of these deteriorated spans relative to the newly replaced girder of the seventh span range between 0.693 and 0.730. The static test of the bridge did find that the flexural rigidity of the old PCI girders was significantly reduced.

### 2.3. Dynamic Test

**Figure 6:**
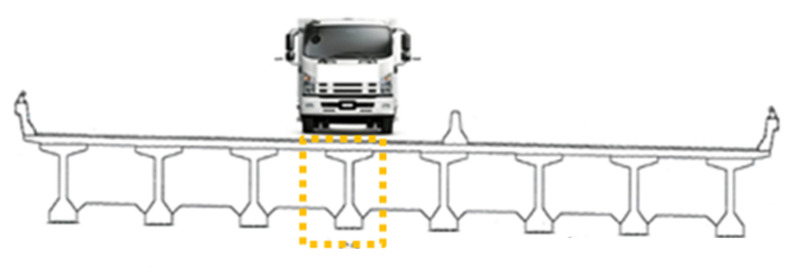
Schematic of the moving vehicle test.

**Figure 7:**
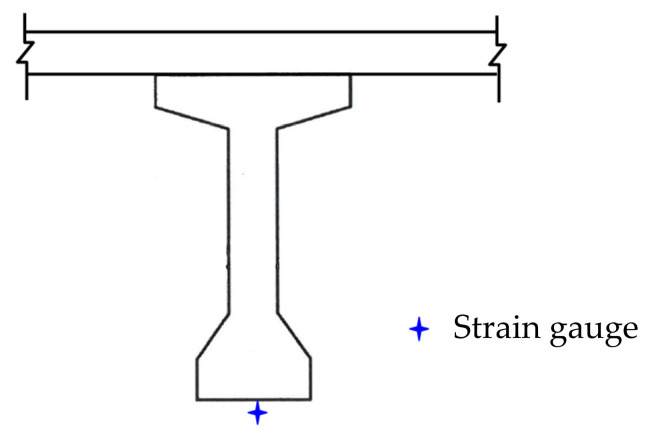
The deployment of the strain gauge for the dynamic test.

In the dynamic test, a 25-ton vehicle passed over each span at a speed of 25 km/h, as displayed in Figure 6. A dynamic strain gauge was placed at the bottom of the midpoint of the girder under the lane where the vehicle passed, as depicted in the yellow box in Figure 7. The sampling frequency of the dynamic strain gauge was 100 Hz. The dynamic strain gauge recorded the strain response caused by the passage of the vehicle over the span.

**Figure 8:**
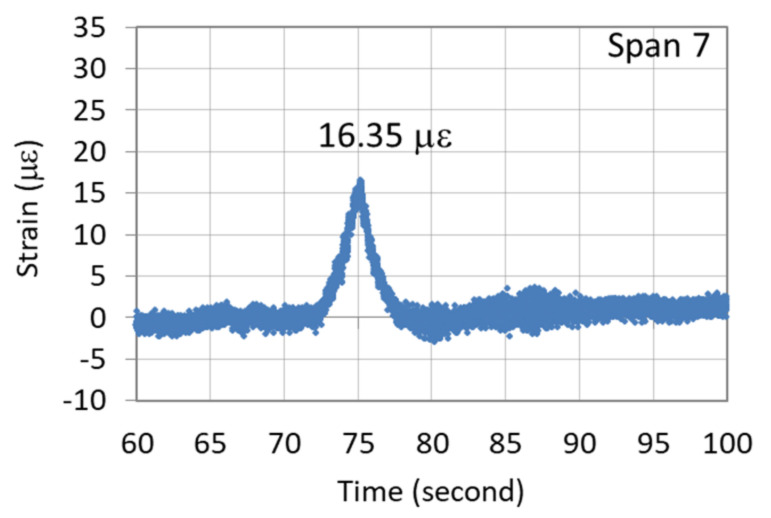
Dynamic strain response of the G5 girder of the seventh span when the vehicle passed over the girder.

**Figure 9:**
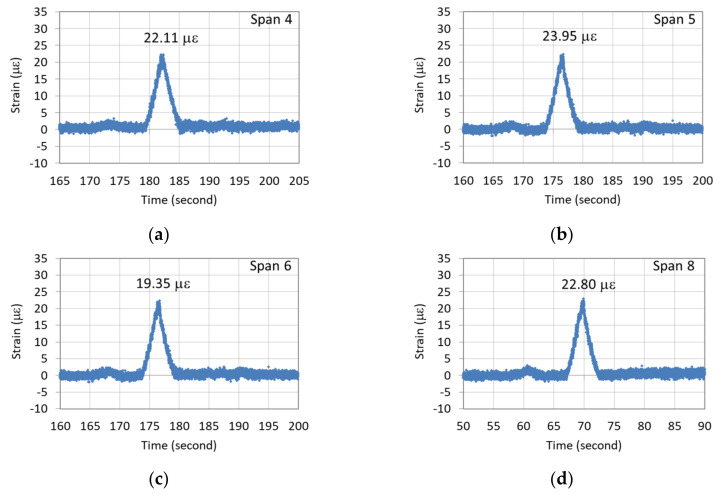
Dynamic strain responses of the G5 girders of the northward spans when the vehicle passed over the girders: (**a**) Span 4; (**b**) Span 5; (**c**) Span 6; (**d**) Span 8.

Figure 8 illustrates the recorded continuous dynamic strain response of the G5 girder of the seventh northward span when the vehicle passed over it. An obvious strain response lasting for approximately 5.8 s was noted for this span, with the maximum vehicle-induced strain response being 16.35 µε. This strain value occurred when the vehicle reached the midpoint of the bridge span. Figure 9a–d depicts the dynamic strain responses caused by the passing of the vehicle at the bottom of the midpoints of the fourth, fifth, sixth, and eighth spans in the northward direction, respectively. The durations of the responses of the aforementioned spans were highly similar to that of the seventh span, which is shown in Figure 8.

As illustrated in Figure 9, the maximum vehicle-induced strain responses of the fourth, fifth, sixth, and eighth spans were 22.11, 23.95, 19.35, and 22.80 µε, respectively. A comparison of Figure 8 and Figure 9 indicates that the dynamic strain response of the newly replaced girder of the seventh span was smaller than those of the fourth, fifth, sixth, and eight spans. The results indicate that the girders of the old bridge spans were considerably deteriorated, and their EI values were significantly lower than that of the girder of the seventh span, which was consistent with the static test results. This means that the dynamic strain responses can serve as an indicator for assessing the degree of bridge deterioration.

## 3. Smart Dynamic Strain Gauge and Edge Computing

### 3.1. Resistance Strain Gauge

**Figure 10:**
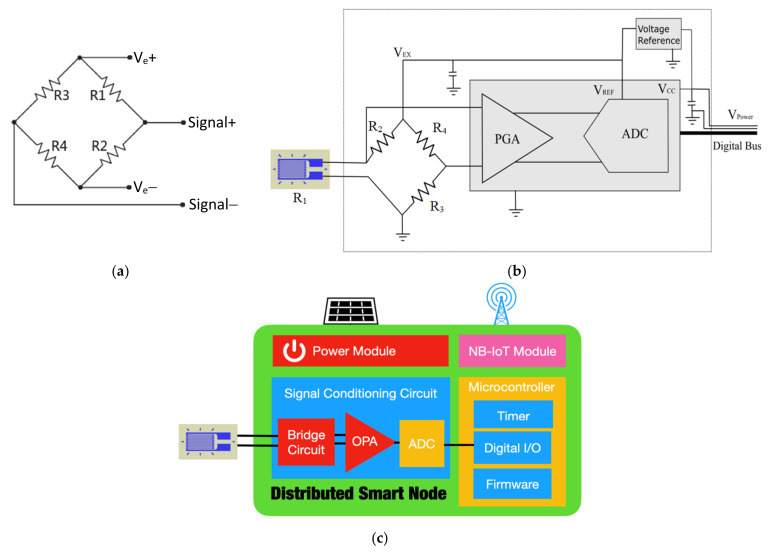
Smart node: (**a**) Wheatstone bridge; (**b**) data acquisition circuit; (**c**) smart node configuration.

The resistance value in a resistance strain gauge changes with the deformation of the measured object. Due to the small change in the resistance (approximately 0.1%), the resistance was converted into a signal with a small relative voltage difference by using a Wheatstone bridge circuit. Figure 10a displays the structure of a Wheatstone bridge. In the figure, R1 is the fixed, yet unknown, resistance to be measured. R2, R3, and R4 are resistors of known resistance.

When the internal resistance ratio (R1/R2) is equal to R3/R4, a fixed excitation voltage between Ve+ and Ve− is applied to the aforementioned bridge. The voltage difference between Signal+ and Signal− at this moment is 0. If R1 is the resistance of the measuring device (e.g., a strain gauge), R1 varies with the deformation of a measured object. At this moment, according to the laws of resistance, the resistance is directly proportional to the length and inversely proportional to the cross-sectional area (Ω ∝ L/A) of the strain gauge. When a linear elastic material is compressed, gauge length decreases and cross-sectional area increases. Thus, R1 decreases, and the balance of the bridge is broken. The voltage of Signal+ is higher than that of Signal−. A positive voltage difference exists between Signal+ and Signal−, and the magnitude of this difference is linearly proportional to the deformation of the measured object. Therefore, the strain of a measured object can be determined from the magnitude of the output voltage difference between Signal+ and Signal−.

Due to the impedance of the wire, the voltage signal is susceptible to loss during transmission in the wires. The longer the transmission line, the higher is the resistance of a wire. Therefore, shorter wire lengths are more suitable for the strain gauge. Consequently, a smart node can be placed adjacent to a strain gauge. This smart node contains a data acquisition unit, and a microcontroller unit (MCU). Figure 10b,c depict the data acquisition unit and the configuration of the smart node, respectively. The data acquisition unit mainly consists of a built-in programmable gain amplifier (PGA) analog-to-digital converter (ADC). The smart node’s microcontroller unit controls the magnification and ADC parameters via serial bus, so that the analog signal transmission wire can be shortened as much as possible. In order to avoid the deviation of the measurement results caused by the power supply drift, the excitation voltage (V_EX_) of the bridge circuit and the reference voltage (V_REF_) of the ADC are designed with rail-to-rail. The V_EX_ and V_REF_ voltages are supplied by a voltage reference IC, which can reduce power consumption and self-heating effects simultaneously. The novel design greatly reduces the wire interference and can reduce V_EX_ and V_REF_ to 2.5V. Calculated by a general 120 ohm bridge circuit, the supply current only needs 20.8 mA. The smart node can perform the analog-to-digital conversion of the strain gauge signal. All the output signals are digital signals; thus, wireless signal transmission can be achieved using the IoT technology. The main advantage of using smart nodes is that the signal transmission is free from the interference of ambient noise, which ensures high signal quality. The sampling resolution and sampling frequency of the smart nodes used in this study were 16 bits and 100 Hz, respectively.

### 3.2. Edge Computing

Monitoring the dynamic strain of a bridge enables the determination of the bridge response caused by the passage of various types of vehicles on the bridge and the mechanical behavior of the bridge, so as to carry out effective safety management. However, continuous dynamic strain monitoring has rarely been performed in practice. In addition to the relatively high cost of dynamic measurement instruments, considerable data must be recorded in dynamic strain monitoring, which results in a high load on the on-site monitoring system, which can lead to high power consumption and system instability; thus, conducting continuous dynamic strain monitoring is difficult.

This study used the technology of edge computing to solve the problem of large amounts of data transmission in existing dynamic strain monitoring systems. Edge computing is based on a distributed computing architecture in which the data computation task of the cloud server is shifted to edge nodes (sensor nodes). The main strength of edge computing is that it reduces the load on the network and servers so that decisions can be made within the edge network, and the latency is reduced. The main advantage of edge computing is that it reduces the load on the network and server. A distributed sensor is equipped with an MCU that contains central processing units, memory, a timer, and various input–output interfaces. It can be placed inside a device; however, the storage capacity of its memory is low, which limits the freedom of MCU firmware development and increases the difficulty of the temporary storage and processing of dynamic data. The MCU used in the study is Texas Instrument MSP430. The MCU’s clock is synchronized with the Network Time Protocol (NTP) server every hour.

**Figure 11:**
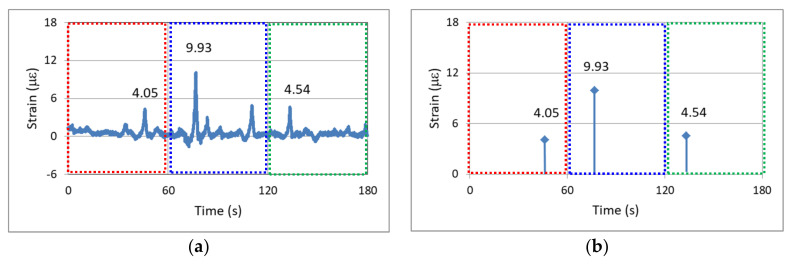
Dynamic strain of the investigated bridge: (**a**) continuous data output; (**b**) outputting of a maximum value every minute.

The main concept of edge computing for the SHM of a bridge is explained in the following text. Vehicles passing over a bridge cause the bridge to exhibit transient strain response; therefore, dynamic strain gauges can be installed at the midpoints of a bridge span to monitor the maximum dynamic response caused by the passage of vehicles, which serves as an indicator for the SHM of the bridge. Two methods exist for outputting the data of a dynamic strain gauge. The first method is the traditional method that involves transmitting all the recorded strain data to the server, which then interprets the data. The second method involves processing and interpreting the data at the sensor nodes through edge computing and then transmitting only key data (e.g., only the local relative maximum strain caused by the passage of vehicles over a bridge every minute) to the server. The difference between the aforementioned two data output and analysis methods is illustrated in Figure 11.

Figure 11a displays the continuous output data of dynamic strain for a duration of 180 s (3 min). In this figure, dynamic strain responses of different amplitudes caused by vehicles passing over the bridge are clearly discernible. As depicted in Figure 11a, 100 data entries were acquired per second, and the continuous output data comprised 18,000 entries.

The second output method only requires the outputting of key data within a certain time range. For example, for the dynamic strain data depicted in Figure 11a, the edge computing outputs only the key data on the occurrence time and magnitude for the local relative maximum strain response per minute (e.g., magnitudes of 4.05, 9.93, and 4.54 µε for the first, second, and third minutes, respectively) as illustrated in Figure 11b. The number of output data entries required in 3 min is reduced from 18,000 to only 3. If the data duration is extended to 1 day, 8,640,000 and 1440 data entries must be output per day when using the first and second data output methods, respectively. Thus, the second data output method allows the monitoring system to operate easily and stably. The above-mentioned edge computing was performed by using the firmware installed in the sensor node.

The basic algorithm of edge computing was to find the local maximum and minimum values of the vehicle-induced strain data by adopting the sliding window and moving average methods. The vehicle-induced strain can then be obtained by subtracting the local maximum value from the local minimum value. The comparison process of each vehicle-induced strain within 1 min can find the relative maximum strain per minute, which is the so-called key data in this study.

**Figure 12:**
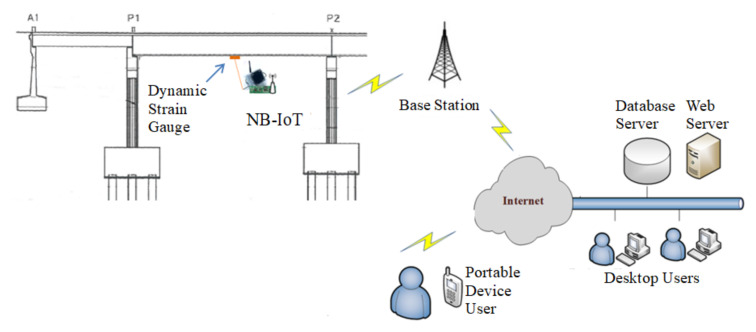
Schematic of the monitoring architecture of the wireless dynamic strain gauge based on NB-IoT that was used in this study.

Since the amount of dynamic strain data to be transmitted is considerably reduced through edge computing, an LPWAN NB-IoT communication module can be used for the wireless transmission of data by the monitoring system. Figure 12 displays the monitoring architecture of the on-site NB-IoT-based dynamic strain gauge used on the investigated bridge. This strain gauge, which was installed at the midpoint of the span of the prestressed girder, contained an NB-IoT communication module that could directly transmit measurement results to the base station of a telecommunications company and then to the Internet. This type of monitoring system can be easily installed and maintained.

In the dynamic strain monitoring system used in this study, a dynamic analog-to-digital converter, an ultra-low-power microcontroller, edge computing firmware, backup memory, and communication technology are combined and integrated to create an ultra-low-power dynamic sensor network system. The excitation voltage of the bridge circuit is 2.5 V and the equivalent resistance of the bridge is 120 ohms, so the current during continuous measurement is 20.8 mA, and the power consumption is 52 mW. The average current consumption of NBIoT transmission every 10 min is 0.83 mA, which leads to an average power consumption of 2.74 mW when the supply voltage of NBIoT is 3.3 V. The power consumption of the microcontroller and the signal processing circuit is about 1.3 mW and 1.4 mW, respectively. Therefore, when the sensing nodes of this system function in combination with its communication nodes, the power consumption of each sensing node is less than 60 mW.

**Figure 13:**
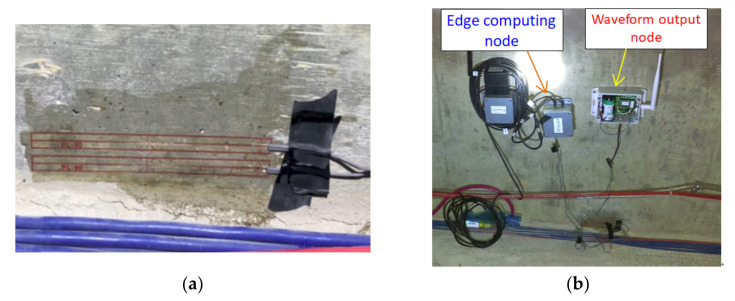
Verification of the functionality of the smart nodes of the wireless dynamic strain gauge: (**a**) strain gauge installation; (**b**) two smart nodes used in the functionality verification.

To verify the functionality of the smart nodes installed on site for edge computing, two smart nodes designed with wireless dynamic strain gauges were simultaneously installed in a prestressed box girder bridge, as displayed in Figure 13. One of these nodes outputted continuous strain waveforms, and the other node used edge computing to only output the maximum strain response caused by passing vehicles per minute.

**Figure 14:**
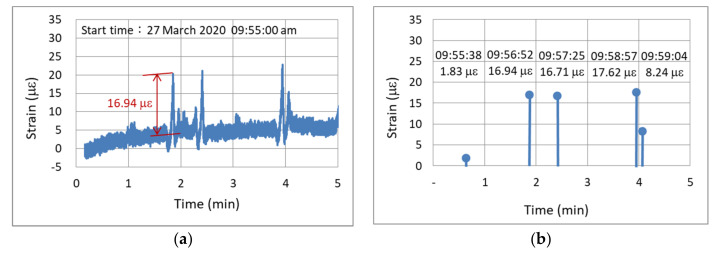
Results of dynamic strain verification: (**a**) output of continuous waveforms; (**b**) output of edge computing.

Figure 14a displays an output signal of the node that outputted continuous strain waveforms. This signal represents the strain data that were continuously output for 5 min between 09:55:00 and 10:00:00 on 27 March 2020. The continuous waveform contains 30,000 data entries. Figure 14b depicts the maximum vehicle-induced strain response per minute that was output by the smart node with edge computing. In edge computing, the sliding window and moving average methods were used to determine the local maximum and minimum values of the vehicle-induced strain data. The aforementioned figure only contains five data entries. A comparison between Figure 14a,b indicates that although the node that used edge computing output only five dynamic strain values for a period of 5 min, it correctly recorded the magnitude and occurrence time of the maximum dynamic strain in each minute. For example, the relative maximum strain value of 16.94 µε at the second minute in Figure 14b corresponds to the amount of change indicated by the red font in Figure 14a. The verification results show that edge computing can effectively and correctly output the key data per minute; thus, edge computing can reduce the load and enhance the performance and stability of the monitoring system.

## 4. On-Site Application of New SHM Method for the Prestressed Bridge

Based on the dynamic test results and the edge computing technology presented in the previous sections, a new SHM method for the PCI girder bridge is proposed. The time-domain dynamic strain response was used to determine the relative strain change caused by the passage of a vehicle in a few seconds, thereby ignoring the temperature effect. This makes the signal analysis of the proposed new SHM method relatively simple, straightforward, and effective.

**Figure 15:**
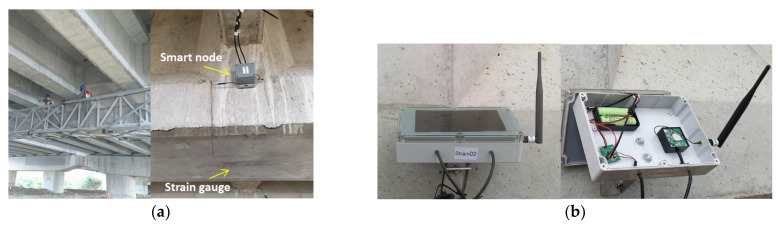
Dynamic strain monitoring device: (**a**) on-site installation; (**b**) solar charging panels with rechargeable batteries.

The bridge mentioned in Section 2 was selected as the location for the on-site application of the new SHM method. In this application study, wireless dynamic strain gauges were installed at the bottom of the midspans of the fifth and seventh northward spans of the simply supported PCI girders (Figure 7). Figure 15 displays a photograph of the installation of the wireless dynamic strain gauges. The use of the edge computing technology with an NB-IoT communication module resulted in the installed devices having low power consumption. Only 6 W solar charging panels with rechargeable batteries were required for the operation of these devices.

**Figure 16:**
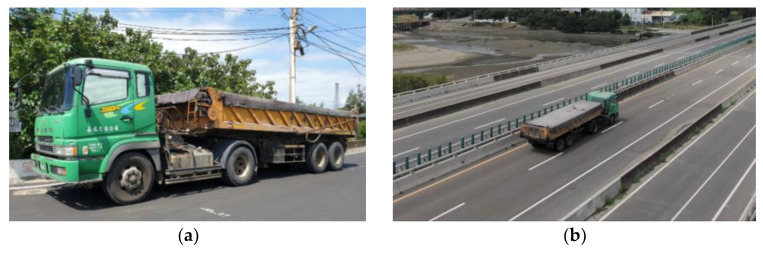
Vehicle loading test: (**a**) image of the vehicle, which weighed 38.46 metric tons, and (**b**) photo of the vehicle when it moved on the bridge.

**Figure 17:**
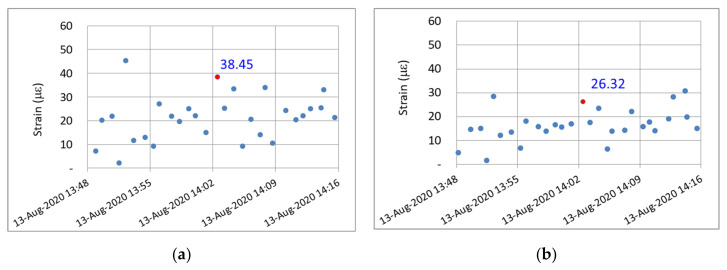
Vehicle loading test: strain responses of the (**a**) fifth and (**b**) seventh spans.

To obtain the dynamic strain response of the PCI girders, a 38.46-ton dump truck was used to conduct a moving vehicle test. The dump truck passed the fifth and seventh spans at 14:02 on 13 August 2020. Figure 16a,b shows the vehicle used in the vehicle load test and this vehicle moving at a speed of 80 km/h, respectively. Figure 17a,b displays the dynamic strain responses detected during the vehicle load test for the fifth and seventh spans, respectively. Dynamic strain responses of 38.45 and 26.32 µε (marked by the red dot) were observed at 14:02 due to the passing of the 38.46-ton test vehicle for the fifth and seventh spans, respectively. In other words, the vehicle weights required to induce unit strain in the fifth and seventh spans were determined to be 1.00 and 1.46 metric tons/µε, respectively. Thus, the flexural rigidity of the newly installed PCI girder of the seventh span was approximately 1.46 times higher than that of the deteriorated PCI girder of the fifth span.

**Figure 18:**
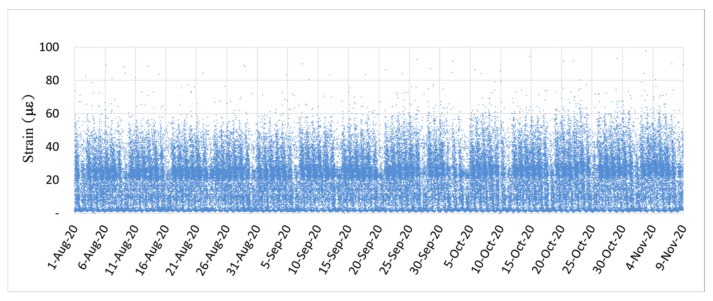
Dynamic strain response caused by the passage of vehicles over the fifth span of the investigated bridge.

**Figure 19:**
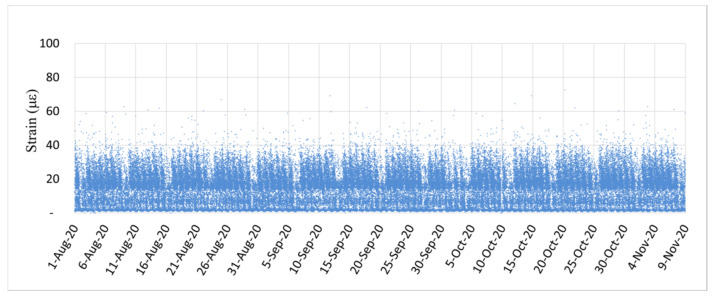
Dynamic strain response caused by the passage of vehicles over the seventh span of the investigated bridge.

Figure 18 and Figure 19 illustrate the dynamic strain responses detected at the fifth and seventh spans, respectively, of the investigated bridge from 1 August to 9 November 2020. Both figures exhibit repetitive dynamic strain responses caused by the passing of vehicles on the bridge on working days. Thus, on working days, the daily traffic volume was rather stable. Considerably fewer instances of high dynamic strain were observed on weekends (indicated by the blank intervals) than on weekdays because there was less heavy-vehicle traffic on weekends. The dynamic strain response of the PCI girder bridge at its seventh span (Figure 19) was smaller than that at its fifth span (Figure 18), which indicates the higher flexural rigidity of the bridge structure. Moreover, Figure 18 and Figure 19 show that the dynamic strain responses of the two spans were stable, which indicates that no significant structural deterioration occurred during the monitoring period. When the monitored dynamic strain response has a tendency to magnify, attention should be paid to the potential prestress loss or other deterioration behaviors of the bridge.

## 5. Discussion

This section discusses the major achievements, limitations and challenges based on the research results presented in this paper.

First, the major achievements of this study include verifying that the flexural rigidity of deteriorated PCI girders can be evaluated by performing constant-weight moving vehicle test, proposing that the vehicle-induced dynamic strain responses can serve as an indicator for assessing the degree of bridge deterioration, and developing a smart node with edge computing functions to avoid transmission of massive data generated through dynamic measurement. It is worth mentioning that the dynamic strain response caused by the passage of a vehicle needs just a few seconds, so that the temperature effect can be ignored. This makes the signal analysis of the proposed new SHM method relatively simple, straightforward, and effective.

However, the bridges investigated in this paper are PCI-girder bridges. Further studies are needed to determine whether the proposed SHM method is still applicable to other types of bridges, such as prestressed concrete box-girders.

Although the monitoring method proposed in this study can improve the measurement benefit of vehicle-induced dynamic strain in terms of processing data volume, the biggest challenge is the development of firmware for edge computing at smart nodes because the processing clock of the microcontroller is only 1 MHz and RAM is only 2 kBytes. In addition, the question of how to apply the measured vehicle-induced dynamic strain to bridge safety management is an important and difficult challenge.

## 6. Conclusions

In this study, static and dynamic tests were conducted on site on a PCI girder bridge. Because of severe deterioration of the seventh northward span, the PCI girder of this span was replaced by new PCI girders, which could serve as the control group in the vehicle loading test. Moreover, this study developed a smart dynamic strain gauge node with edge computing functions. This node only transmitted the relative maximum dynamic strain data per minute, which considerably reduced the original 6000 output data entries per minute to only one output data entry per minute; thus, this node can be paired with an NB-IoT wireless communication module to develop a high-performance dynamic strain sensor capable of independent operation.

The following conclusions are drawn from the results of this study:

1. The results of the static test showed that the EI/M ratios of the deteriorated spans relative to the newly replaced girder of the seventh span range between 0.693 and 0.730. The static test of the bridge did find that the flexural rigidity of the old PCI girders was significantly reduced.
2. The results of the dynamic test indicated that the relative maximum dynamic strain of a newly installed PCI girder was significantly smaller than that of old and deteriorated PCI girders. Thus, the flexural rigidity of the old and deteriorated PCI girder was significantly smaller than that of the newly installed PCI girder.
3. Based on the dynamic test results and the edge computing technology presented in the study, a new SHM method for the PCI girder bridge is proposed. The time-domain dynamic strain response was used to determine the relative strain change caused by the passage of a vehicle in a few seconds, so that the temperature effect can be ignored. This makes the signal analysis of the proposed new SHM method relatively simple, straightforward, and effective.
4. The on-site monitoring results showed that under routine traffic conditions, the dynamic strain response of the new PCI girder was smaller than that of the deteriorated PCI girder. The dynamic strain responses of the two spans were stable under routine traffic conditions. This indicates that no significant structural deterioration occurred during the monitoring period. When the monitored dynamic strain response has a tendency to magnify, attention should be paid to the potential prestress loss or other deterioration behaviors of the bridge.

This paper introduces the application of smart dynamic strain gauge nodes with edge computing functions in bridge dynamic strain monitoring, which solves the problem of massive data transmission and makes the field application of dynamic strain monitoring more feasible. Subsequent further research can include monitoring and tracking the neutral axis position of bridges, applications in bridge weight in motion, and the combination of some computational intelligence techniques such as model updating using evolutionary computation or data-driven machine learning tools.

## Figures and Tables

**Table 1:** Static test results.

Span No.	Bottom Strain ε_b_ (10^−6^)	Upper Strain ε_u_ (10^−6^)	h (cm)	EI/M = h/(ε_b_−ε_u_) (10^6^ cm)	(EI/M) Ratio
Span 4	26.85	−4.25	138.5	4.453	0.698
Span 5	26.78	−4.90	140.0	4.419	0.693
Span 6	25.48	−2.19	134.5	4.861	0.762
Span 7	22.89	1.65	135.5	6.379	1.000
Span 8	27.28	−3.33	142.5	4.655	0.730

## Data Availability

Some or all data, models, or code that support the findings of this study are available from the corresponding author upon reasonable request.
